# Diet quality determines interspecific parasite interactions in host populations

**DOI:** 10.1002/ece3.1167

**Published:** 2014-07-15

**Authors:** Benjamin Lange, Max Reuter, Dieter Ebert, Koenraad Muylaert, Ellen Decaestecker

**Affiliations:** 1 Laboratory Aquatic Biology, Science & Technology-Kulak, KU LeuvenKortrijk, 8500, Belgium; 2 Department of Genetics, Evolution & Environment, University College LondonLondon, WC1E 6BT, UK; 3 Zoological Institute, University of BaselBasel, CH-4051, Switzerland

**Keywords:** *Daphnia magna*, disease dynamics, food quality, host population demography, interspecific parasite competition, multispecies infections, parasite ecology, unicellular gut parasite, white bacterial disease

## Abstract

The widespread occurrence of multiple infections and the often vast range of nutritional resources for their hosts allow that interspecific parasite interactions in natural host populations might be determined by host diet quality. Nevertheless, the role of diet quality with respect to multispecies parasite interactions on host population level is not clear. We here tested the effect of host population diet quality on the parasite community in an experimental study using *Daphnia* populations. We studied the effect of diet quality on *Daphnia* population demography and the interactions in multispecies parasite infections of this freshwater crustacean host. The results of our experiment show that the fitness of a low-virulent microsporidian parasite decreased in low, but not in high-host-diet quality conditions. Interestingly, infections with the microsporidium protected *Daphnia* populations against a more virulent bacterial parasite. The observed interspecific parasite interactions are discussed with respect to the role of diet quality-dependent changes in host fecundity. This study reflects that exploitation competition in multispecies parasite infections is environmentally dependent, more in particular it shows that diet quality affects interspecific parasite competition within a single host and that this can be mediated by host population-level effects.

## Introduction

Parasites play a key role as consumers in our environment (Dobson et al. 2008), *for example* in food webs (Lafferty et al. 2006) and during species invasions (Johnson and Thieltges 2010; Dunn et al. 2012). Their commonness and significance for ecosystems is gaining more and more attention (Hudson et al. 2006; Holdo et al. 2009; Sato et al. 2012). It is frequently observed that hosts are infected by multiple parasite species (Ebert 2005; Poulin 2007; Schmid-Hempel 2011). Multispecies infections co-infecting the same host individual may be independent, but parasites can also interact in a synergistic or antagonistic way, modifying each other's effect on the host (Hughes and Boomsma 2004; Pedersen and Fenton 2007; Graham 2008; Thumbi et al. 2013). Beyond interactions within individual hosts, synergistic and antagonistic parasite interactions can also be observed within host populations (Dobson 1985; Lello et al. 2004; Bell et al. 2006; Graham 2008; Telfer et al. 2008, 2010; Mideo 2009).

Interacting parasites can affect each other's survival (de Roode et al. 2004; Alizon et al. 2013) and virulence (Balmer et al. 2009). The strength of the interactions depends on many different factors, such as host behavior (Bush and Malenke 2008), the infection dose to which the host is exposed (Fellous and Koella 2009), or the order in which multiple infections occur (Lohr et al. 2010; Hoverman et al. 2013). Besides these intrinsic host properties, parasites can be largely affected by the environmental factors to which their hosts are exposed (Wolinska and King 2009; Karvonen et al. 2010; Pech et al. 2010; Vale et al. 2011). Evidence for the relevance of these factors, with respect to single species infections, is amply present in a number of different systems: bacteria–phage interactions (Forde et al. 2004), plant–pathogen interactions (Laine and Tellier 2008; Burdon and Thrall 2009), and invertebrate–pathogen interactions (McKenzie and Townsend 2007; Garbutt et al. 2014; Hall et al. 2013; for an overview see Schmid-Hempel 2011). Nevertheless, with the exception of temperature and humidity (see Thomas et al. 2003; Malenke et al. 2011), the effects from environmental factors on multispecies parasite interactions have been largely neglected, even though ecological constraints of host resources are considered to play an important role (Pedersen and Fenton 2007; Bukovinszky et al. 2009; Sarfraz et al. 2009).

Contrary to food quantity (amount in mg Carbon/L), food quality is usually defined by elemental ratios, such as low carbon-to-nitrogen (associated with high protein quality, Anderson et al. 2004) or low carbon-to-phosphor ratios (largely associated with P-rich ribosomal RNA production, Elser et al. 2000) or by particular lipids, such as PUFAs or sterols (Martin-Creuzburg and Von Elert 2009). In vertebrates, parasites mostly suffer from both a higher quantity or a better quality diet of the host, based on increased host immune system activity (Wiehn and Korpimäki 1998; Lochmiller and Deerenberg 2000). Reversely, monospecies parasite infections of invertebrate hosts often benefit from an increase of host food quantity (McKenzie and Townsend 2007; Seppälä et al. 2008; Civitello et al. 2013), although quantity levels that are too high can also terminate parasite epidemics (Dallas and Drake 2014). To date, however, possible effects of host diet on multispecies parasite infections remain elusive. We here focus on this research question, evaluating the environmental factor of host diet quality, as it is a determining and ubiquitous factor for heterotrophic animal hosts, which has shown to affect growth and virulence of individual parasite species (Hall et al. 2009; Choisy and de Roode 2010). We consider diet quality as a difference in the presence of particular nutrients that are important for the growth and reproduction of a heterotrophic animal and observed the difference in host population density as well as parasite fitness in different food quality environments.

In natural ecosystems, parasites live in host populations and transmission between different host individuals is usually an essential fitness parameter that determines parasite epidemiology and virulence (Dieckmann 2002; Poulin 2007). Despite the importance of host to host transmission, most studies investigating infected host populations are based on within-host model systems, and from these individual host studies, one extracts expectations for host population-level effects of parasitism (Choisy and de Roode 2010). In particular, parasite transmission between host individuals in a population may be relevant for understanding multispecies parasite interactions. One of the few studies, where a host population model was used, found fundamental differences in host population dynamics in the monoparasitic *Plodia interpunctella* granulovirus system, due to changes in food quality (McVean et al. 2002). While a range of studies confirmed that even parasite communities can differ depending on environmental conditions (Rohde and Heap 1998; Nunn et al. 2005), these descriptive analyses could not uncover interactions between specific parasites (Rigaud et al. 2010; Johnson and Buller 2011). For these reasons, we have chosen to study host diet quality effects on multispecies parasites interactions experimentally, explicitly allowing host population-level-mediated effects, while controlling the identity of parasite species.

Here, we use the established experimental invertebrate model organism *Daphnia magna* that is infected by a multitude of microparasites, most of them transmitting horizontally between individuals in the host population, and many of which are known and their life cycle described in the literature (Decaestecker et al. 2005; Ebert 2005; Wolinska et al. 2009; Duffy et al. 2010; Jansen et al. 2010). Earlier experimental studies, investigating uninfected *D. magna* individuals, suggested a possibly strong effect of food quality on *Daphnia* population dynamics, because somatic growth depends on nutrient and cholesterol availability, while reproduction depends on PUFA availability (Wacker and Martin-Creuzburg 2007; Martin-Creuzburg and Von Elert 2009). In *Daphnia*, food quality has also been shown to affect parasitism. In particular, the relative availability of nutrients and the presence of PUFAs can affect *Daphnia–*parasite interactions (Frost et al. 2008; Hall et al. 2009; Schlotz et al. 2013), but effects on population level have rarely been studied and are not straightforward (Aalto et al. 2014; Dallas and Drake 2014). These findings allow us to target the availability of sterols and PUFAs, by using different food choices with variable sterol and PUFA contents, and here we measure its direct effect on population density rather than on host individuals. We chose two parasites for this study, the unicellular gut parasite (UGP) and the parasite that causes white bacterial disease (WBD). These two parasites differ strongly in their effect on *D. magna* mortality and fecundity (Ebert et al. 2000; Ebert 2005) and co-occur in natural host populations (Decaestecker et al. 2005; Ebert 2005). The aim of this work was to investigate the role of host diet quality in parasitized *D. magna* populations, comparing monospecies- versus multispecies-infected populations. More specifically, the objectives of the study were to determine the effect of diet quality on: (1) competition between two co-infecting parasite species; (2) single- and multiple-infected host population densities; and (3) host population demography, particularly the density of adult/juvenile hosts.

## Materials and Methods

### Host-parasite system

To study food quality effects on parasite interactions, we used the water flea *D. magna* STRAUSS and two of its (endo-)parasites. Monoclonal offspring of a female *Daphnia* were used in the experiments. The female was collected from a Belgian pond (situated in the coastal zone, 51°21′25″N, 3°20′34″E) in May 2010. Two *Daphnia* parasites were isolated from the same pond: White bacterial disease (WBD), also known as white fat cell disease, is a highly virulent horizontally transmitting parasite that infests the fat tissue of its host (Ebert 2005; Coopman et al. 2014). During early infection, stages of the parasite are not yet visible to the naked eye. After several days, the host's fat cells start to have a whitish-green shine and the infection is clearly visible. In earlier studies, infected animals died within 3 weeks postinfection (Ebert et al. 2000; Van De Bund and Van Donk 2002; Decaestecker et al. 2003). The second parasite used here is the unicellular gut parasite (UGP), also described before as Micro1 in Decaestecker et al. (2003, 2005). It is a horizontally transmitting microsporidian parasite that can be found primarily at the end of the host gut and is characterized by low virulence, a very small parasite-induced increase in mortality (Refardt and Ebert 2012).

### Culture conditions

For our experiments, food treatments differing in cholesterol and PUFA availability were chosen. Strains of the algae *Scenedesmus obliquus* (SAG 276-3a; culture collection of algae, University of Göttingen in Germany), *Chlamydomonas reinhardtii* (SAG 77.81), and *Cryptomonas* sp. (SAG 26.80) were grown in modified WC medium with vitamins at 20 ± 1°C (Guillard 1975). In particular, with respect to PUFAs, *S. obliquus* and *C. reinhardtii* are described as low food quality, while *Cryptomonas* sp. is considered to be high food quality (Weers and Gulati 1997; Martin-Creuzburg and Von Elert 2009; Basen et al. 2011). Prior to all experiments, the animals were fed with the green alga *S. obliquus* for several months to standardize food conditions for all host populations (Taipale et al. 2011). For the experiments, food quality was standardized for organic Carbon according to Bird et al. (2011) so that there is no quantitative but only a qualitative difference between food treatments. Experiments were conducted under a constant light/dark cycle (16:8 h) at a temperature of 20 ± 1°C. The artificial *Daphnia* medium ADaM (modified by using only 5% of the recommended SeO_2_ concentration) was used for all cultures (Klüttgen et al. 1994).

### Experimental setup

To test the influence of food quality on parasite interactions, a host population experiment was conducted using ten replicated populations per treatment. Infected or uninfected (control) populations of ten hosts (five adults, five juveniles) were set up in beakers containing 300 mL of medium. The populations were either fed high or low food quality three times per week (2 mg Carbon/L). After 5 weeks, the treatment was increased to 4 mg Carbon/L. Medium was changed in weekly intervals, and adult and juvenile *Daphnia* were counted. Dead animals were not transferred to the new medium because spore transmission and bacterial growth might influence the host–parasite dynamics (Ebert et al. 2000). The experiment lasted 10 weeks, which equals about five host generations. During this time, WBD-exposed populations were checked visually once per week for WBD infections. After 10 weeks, five adult host individuals were randomly chosen from each surviving host population. Then, *Daphnia* body length (as in Ranta et al. 1993) and amount of UGP spores (as in Decaestecker et al. 2005) were assessed for each individual in the UGP treatments. All animals surviving the other treatments were checked for UGP infection at the end of the experiment. Absence of WBD infections, in the controls and solely UGP-exposed populations, was verified by weekly observations during the experiment.

### Data analysis

In the parasite fitness part of this study, we were primarily interested in the effects of co-infection and food quality. We analyzed our experimental data on parasite fitness using an analysis of variance (ANOVA). The full model contained the following main explanatory variables: (1) fixed effects of ‘host body size’ (continuous), (2) ‘food’ (nominal) quality, or (3) ‘WBD’ infection (nominal: singly infected population versus co-infected population with WBD & UGP), and the interactions between these explanatory variables. The response variable was the average number of UGP spore clusters for each host population. We followed backward elimination of nonsignificant terms, and thus removed the interactions between host body size and food as well as host body size and WBD.

In the host population part of this study, the mean host density of weeks eight to ten (equilibrium density) was used for analysis. To that purpose, the arithmetic mean of population density of adults, juveniles, or both together were compared with the mean population density of uninfected populations within the same food and parasite treatment combination, using a Wilcoxon two-sample test. Because several populations became extinct after 10 weeks of WBD treatment, a chi-square test for host extinctions comparing parasite to uninfected treatment was used instead of the Wilcoxon two-sample test. Generally, statistical significance was accepted at the *α* < 0.05 level. To counteract type I errors, the results of the Wilcoxon two-sample test were Bonferroni corrected and thus only accepted at the *α* < 0.003 level. All analyses were performed in R (R Development Core Team 2009).

## Results

### Effects of host diet and co-infection on UGP fitness

**Figure 1:**
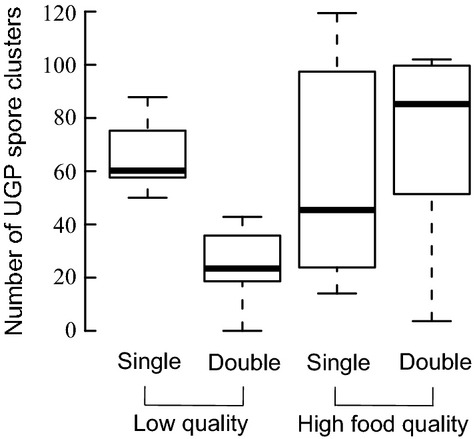
Box-Whiskers plot showing the mean number of UGP (unicellular gut parasite) spore clusters after 10 weeks in surviving host populations in UGP infected (single) or co-infected (UGP & WBD (White bacterial disease), double) populations. The outer *x*-axis shows the feeding regime of the host populations (low or high food quality). Mean UGP spore cluster numbers were calculated from randomly selected adult individuals for each host population [*n* = 5].

Parasite fitness was measured by assessing the number of UGP spore clusters in *Daphnia*. The number of UGP clusters was affected by interactions of UGP with WBD and food quality (Table 1; Fig. 1). The mean number of UGP clusters was higher in hosts of single-UGP-infected populations than in hosts of co-infected populations in the low food quality treatment (single infected: 66.38 ± 13.42 (mean ± SD); co-infected: 26.11 ± 13.65; Welch's test, *t*_16.00_ = 6.31, *P* < 0.0001). In contrast, no difference in UGP cluster number between the single and co-infected hosts was observed in the high food quality treatment (single infected: 55.70 ± 39.43; co-infected: 72.28 ± 34.82; Welch's test, *t*_15.80_ = −0.95, *P* < 0.36).

**Table 1:** ANOVA results of population experiment for the effects of host body size, food quality treatment, and WBD (white bacterial disease) infection on the number of clusters of UGP (unicellular gut parasite) after 10 weeks. Five host individuals per population were measured.

Explanatory variable(s)	df	*F*	*P*
Size	1, 31	9.11	0.006
Food	1, 31	4.12	0.051
WBD	1, 31	3.52	0.071
Food × WBD	1, 31	4.47	0.043

### Effects of host diet quality and co-infection on host population dynamics

**Figure 2:**
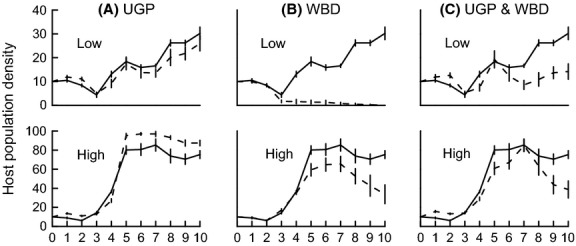
Population dynamics of uninfected *Daphnia* populations (continuous line, means of 10 replicate populations with SE) and infected *Daphnia* populations (dashed line, means of ten replicate populations with SE). Infected populations carried (A) UGP (unicellular gut parasite), (B) WBD (white bacterial disease), or (C) UGP and WBD together. The populations were kept under a low food quality (Low) or high food quality (High) treatment. For each food quality, the same uninfected control populations were plotted for all parasite treatments.

Infected host populations differed in the rate of population extinction. None of the control populations became extinct. Most singly WBD-infected populations went extinct, while all other parasite treatments showed intermediate host population extinction rates (Table 2). Higher food quality led to a higher host equilibrium population density (Fig. 2). The equilibrium density of the UGP-infected populations did not differ from the density of the control populations (neither at high, nor at the low food quality treatment). However, the age structure in UGP-infected populations showed significantly more juveniles than the control populations at high, but not at the low food quality (Table 2). *Daphnia* equilibrium population densities in co-infected and WBD-infected populations were half the density of UGP-infected populations under the high food quality treatment (Fig. 2). The equilibrium density of co-infected populations was significantly higher than that of WBD-infected populations at low (co-infected: 12.93 ± 0.18; WBD-infected: 0.27 ± 0.18; Welch's test, *t*_9.09_ = 4.84, *P* < 0.001), but not at high food quality (co- infected: 48.50 ± 8.67; WBD-infected: 43.00 ± 9.11; Welch's test, *t*_17.69_ = 0.44, *P* < 0.67). *Daphnia* equilibrium density in co-infected populations was also intermediate compared to the density of the UGP-infected populations under the low food quality treatment (Fig. 2).

**Table 2:** Summary of population experiment results with two different food quality and two parasite species treatments. Shown are relative densities of infected host populations statistically compared with uninfected populations in the same food and parasite treatment combination.

Food treatment	Parasite treatment	Host extinctions after 5/10 weeks	Equilibrium density of adult and juvenile hosts (±SE)	Equilibrium density of adult hosts (±SE)	Equilibrium density of juvenile hosts (±SE)
Low quality food	UGP	0/1	0.82 ± 0.11	0.82 ± 0.11	0.82 ± 0.12
	WBD	6/10***	NA	NA	NA
	UGP & WBD	0/1	0.47 ± 0.09***	0.36 ± 0.08***	0.52 ± 0.11
High quality food	UGP	0/0	1.22 ± 0.03	0.62 ± 0.04***	1.77 ± 0.05***
	WBD	0/4***	0.59 ± 0.12	0.47 ± 0.11	0.70 ± 0.16
	UGP & WBD	0/2	0.66 ± 0.12	0.47 ± 0.07***	0.84 ± 0.19

WBD, white bacterial disease; UGP, unicellular gut parasite.

*** *P* < 0.001.

## Discussion

We investigated the effect of host resources, mediated by diet quality on the interspecific association between two parasites infecting the freshwater crustacean *D. magna*. In particular, infected or uninfected experimental host populations, instead of host individuals, were treated with high or low food quality and resulting population dynamics were analyzed. Our results reflect interspecific parasite interactions and showed that a low-virulent microsporidian species (UGP) suffered from co-infection with a high-virulent bacterial (WBD) parasite at low, but not at high food quality. Similarly, results of earlier studies on within-host competition showed fitness disadvantages for lesser virulent parasites (Gower and Webster 2005; Bell et al. 2006). The disadvantage is explained by competition favouring increased host exploitation and thus increased virulence (Choisy and de Roode 2010). The result could also be attributed to increased host defenses (top-down control; Pedersen and Fenton 2007; Graham 2008; Johnson and Buller 2011), due to the possible presence of co-infecting high-virulence parasites which would strongly activate the host's immune system.

Here, we chose to use a host population-level approach, rather than a host individual approach, as individual-based infection trials exclude host–host interactions and parasite transmission between hosts. These interactions influence the fitness and virulence of parasites in natural populations (Ebert 1998; Schmid-Hempel and Ebert 2003; Woolhouse and Gowtage-Sequeria 2005; Refardt and Ebert 2007). Consequently, competition, which could not be observed in individual host infection trials, might also emerge between parasites as a result of more complicated mechanisms at the host population level (Pedersen and Fenton 2007; Johnson and Buller 2011). In particular, competition may arise due to a change in the host population's demographic structure, which is what we observed here. In our study, higher food quality increased the host fecundity (reflected in a higher juvenile compared to adult host population density), both in the absence and presence of parasitism. This outcome tallies with previous studies, which showed that food quality, more precisely PUFA and sterol availability in general, leads to a higher fecundity of copepods and cladocerans (Arendt et al. 2005; Wacker and Martin-Creuzburg 2007; Martin-Creuzburg and Von Elert 2009). Although parasites can shape host population dynamics (Ebert et al. 2000), the fecundity increase caused by the higher food quality overruled the negative effect of the parasites. Higher food quality led to a higher host offspring production in a straightforward way, but the parasites’ effect on host reproduction is more complex. One effect that may have occurred is that parasite infections lead to fecundity compensation, which is a nonimmunological mechanism that contributes to host defense (Parker et al. 2011; Schmid-Hempel 2011). Fecundity compensation can be realized by inducing earlier host reproduction in infected hosts as a mean to increase its fitness (reproduction shift), and as such may affect host population dynamics (as shown for *Daphnia* parasitism in Chadwick and Little 2005). As increased fecundity can be affected by both food quality and the presence of parasites, it is likely the cause of the interaction between food quality and parasites found in this study. More specifically, the increased fecundity is likely to affect the higher equilibrium population density for the UGP-infected populations compared to the other infected populations at high food quality.

Our results suggest that the host populations were protected against the more virulent WBD by the less virulent UGP under the low food quality treatment. Under low food quality, WBD-infected host populations were less viable than co-infected populations, showing that competition between parasites can be beneficial to the host. It is also at low food quality that we detected a lower UGP growth rate in the co-infected populations. Thus apparently the growth of UGP is more constrained, but at the same time the host is more protected against WBD in the co-infected condition. Similarly, but then at the within-species level, co-infections with different strains of the protozoan parasite *Trypanosoma brucei* have been shown to result in higher host survival (Balmer et al. 2009). The consequences of co-infections with different strains have also been investigated for the parasitic genus *Plasmodium* (Read and Taylor 2001; de Roode et al. 2005; Wargo et al. 2007). Observations of *Plasmodium falciparum* implied competition between strains, leading possibly to protection against genetically distinct *Plasmodium* superinfections (Mercereau-Puijalon 1996; Smith et al. 1999). Between parasite species, especially bacteria, competition and mechanisms preventing superinfections have been shown (Harrison et al. 2008; Hibbing et al. 2010). The results presented here are to our knowledge the first results that show that food quality determines competition between different parasite species and that this may have a beneficial, protective effect for the host population. This result shows that interactions between parasites are not only influenced by host and parasite genotypes (Thomas et al. 2003; de Roode et al. 2004), but also by food quality provided to the host.

There might be a mechanistic explanation for the food quality-dependent interactions between the two parasites we tested here based on their different life cycles and shared resources (Griffiths et al. 2014). UGP causes chronic infection with increased parasite spore production when the host gets older. Thus, UGP depends on long-living host individuals that, once infected, spread an increasing amount of spores until their death (Ebert 2005). In contrast, WBD kills its host relatively fast and is not dependent on long host lifespan (Decaestecker et al. 2003; Coopman et al. 2014). However, a host that is already infected with UGP might be less susceptible to infection or at least less viable as a host for WBD. While in high food quality levels, the negative effects of these parasites on each other might be reduced, in low food quality environments, UGP cannot compensate the WBD-induced death of UGP spreaders anymore, as a result of which WBD has a stronger impact on the host population. Accordingly, in our experiment host population density of co-infected populations increased, compared to singly WBD-infected populations, in low but not in high host diet quality. Because the life history of both parasites depending on the same host populations collide and fitness of UGP can be lowered by presence of WBD, the association between these two parasites can be described as exploitation competition (Dunn 2005; Dunn et al. 2012).

In conclusion, the host population design of this study allowed us to omit the limitations of individual-based infection trials and increased the robustness of the results. We infer that food quality in the environment modifies interspecific competition between parasite species and that this outcome is likely associated with population demographic effects. From these insights, we believe that understanding resource-dependent co-occurrence of different parasite species will help us to better grasp the make-up of natural parasite communities and to gather further insights into disease dynamics. Furthermore, environmental factors, such as changing temperatures, may influence the effect of *Daphnia* nutrition on its reproduction (Pajk et al. 2012) and in consequence *Daphnia–*parasite interactions (Mitchell et al. 2005) and would therefore be interesting additions to any future investigation.

## Data Accessibility

Original experimental data will be stored at Dryad.

## Supporting Information

**Figure S1.** *Daphnia magna* with white bacterial disease. Picture by Joachim Mergeay, Leuven.
