# Supplementary figures and images for: Diet quality determines interspecific parasite interactions in host populations
Source: Ecol Evol. 2014 Jul 15;4(15):3093–102. doi: 10.1002/ece3.1167 (PMC4161182; doi:10.1002/ece3.1167)

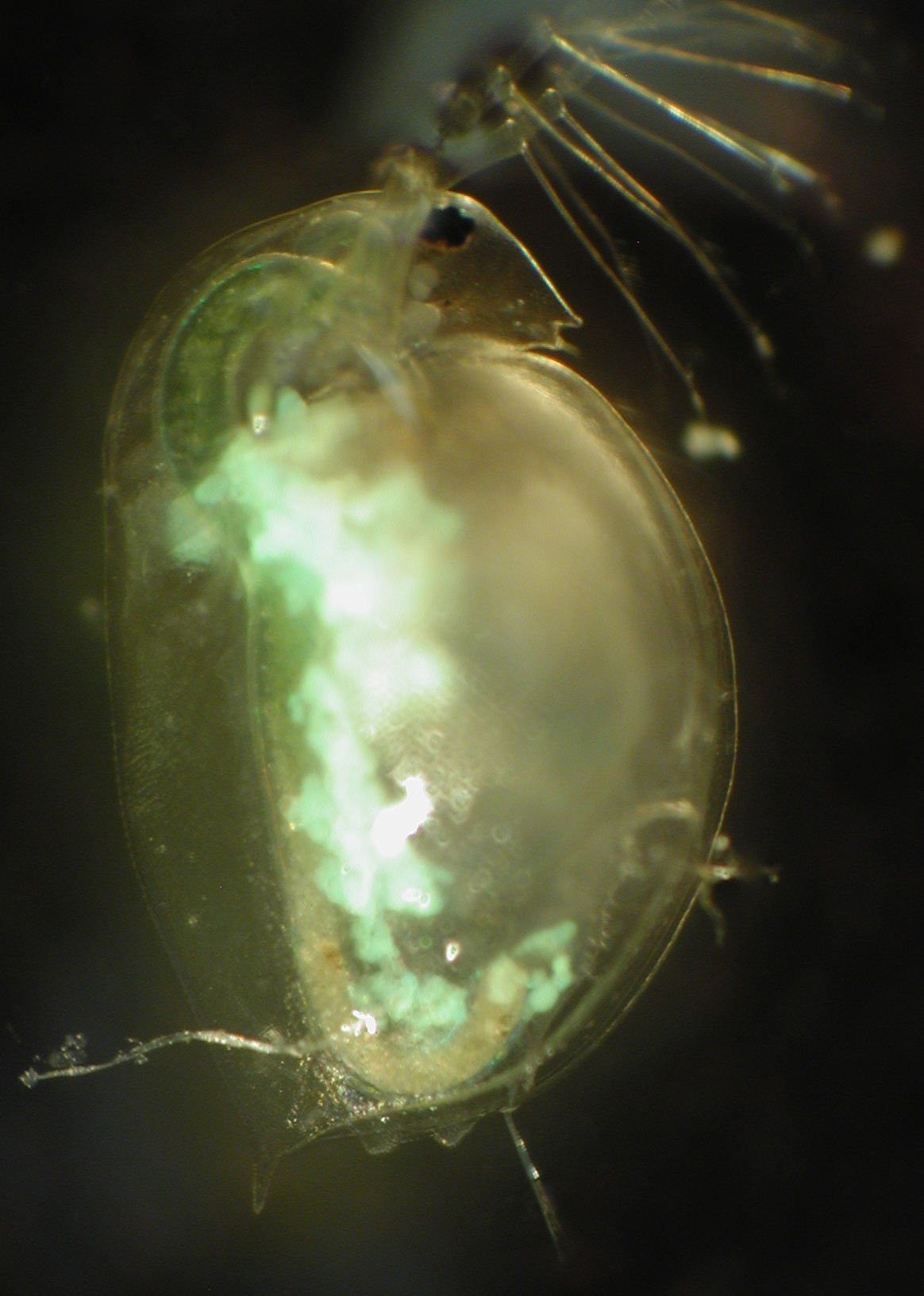

Supplement: Supplementary file 1 — Figure S1. Daphnia magna with white bacterial disease. Picture by Joachim Mergeay, Leuven. [file ece30004-3093-sd1.jpg]
